# Thrombocytopenia-absent radius syndrome: prenatal diagnosis of a rare syndrome

**DOI:** 10.1590/0100-3984.2015.0117

**Published:** 2016

**Authors:** Natália Canhetti Bertoni, Daniela Cardoso Pereira, Edward Araujo Júnior, Luiz Claudio de Silva Bussamra, José Mendes Aldrighi

**Affiliations:** 1 Faculdade de Ciências Médicas da Santa Casa de Misericórdia de São Paulo (FCMSCSP), São Paulo, SP, Brazil.; 2 Escola Paulista de Medicina - Universidade Federal de São Paulo (EPM-Unifesp), São Paulo, SP, Brazil.

**Figure 1:**
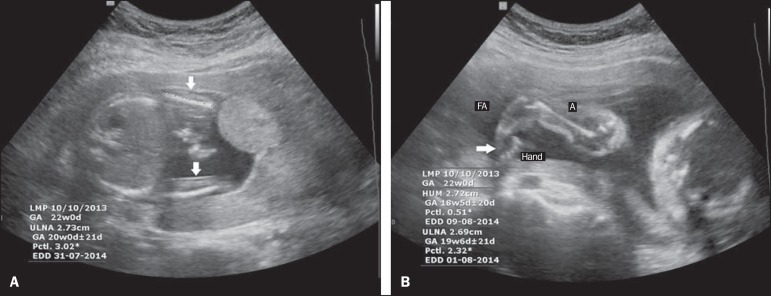
Ultrasound images of a fetus with TAR syndrome, at 21 weeks of gestation. **A:** Axial plane scan at the abdominal circumference measurement level, showing shortening of the ulnae and the absence of radii (white arrows). **B:** Sagittal plane scan at the level of the humeral length measurement, showing internally rotated hands (white arrow). FA, forearm; A, arm.

Dear Editor,

A 32-year-old woman in her third pregnancy was referred for prenatal care because of fetal malformations found on a routine ultrasound. In the second trimester fetal morphology ultrasound scan (conducted at 21 weeks of gestation), the following were identified: mild right pericardial effusion, shortened ulnae, shortened humeri (< 1st percentile for gestational age), and no radii (2nd percentile for gestational age), as shown in Figure 1A; and internally rotated hands, as shown in Figure 1B. There were no alterations in the lower limbs. The fetal biometry was consistent with the gestational age, the estimated gestational weight was 463 g, and the amniotic fluid index was 10.4 cm.

**Figure 2:**
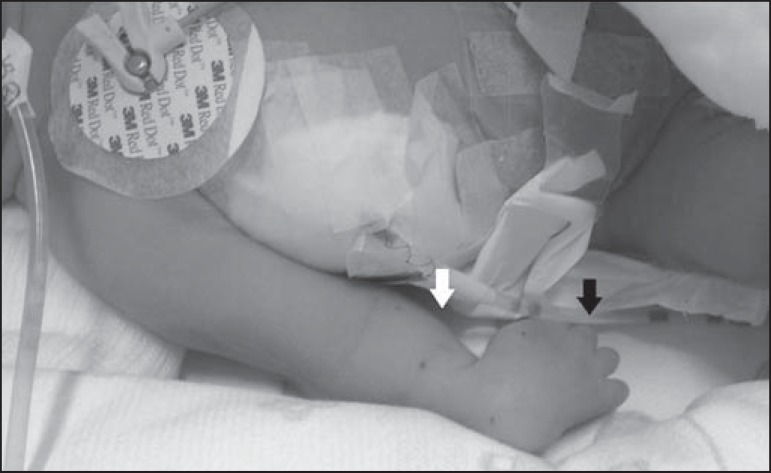
Ultrasound image of the newborn, showing the shortening of the forearm (white arrow) and the internal rotation of the hand (black arrow).

Follow-up ultrasound scans were performed every four weeks. At 31 weeks, the mother went into preterm labor, evolving to normal delivery without complications. The newborn developed respiratory distress, requiring endotracheal intubation and mechanical ventilation. Physical examination revealed deformity of the upper limbs, without other anatomical changes (Figure 2). On the sixth day of life, the ventilation patterns worsened and the infant developed pneumothorax, subsequently evolving to death.

The advent of ultrasound imaging represented a major advance in the prenatal diagnosis of fetal malformations^(1,2)^. The diagnostic criteria for thrombocytopenia-absent radius (TAR) syndrome are bilateral radial agenesis, with preservation of the index finger, and thrombocytopenia. Thrombocytopenia can manifest at any age, from the prenatal period to adulthood^(3)^. It has been reported that TAR syndrome can be accompanied by craniofacial, cardiac, digestive, urogenital, and psychiatric abnormalities, as well as by lactose intolerance^(4)^.

The diagnosis of TAR syndrome is based on ultrasound findings and fetal blood sampling by cordocentesis to determine the number of platelets. The diagnosis can be confirmed by a genetic test using fetal cells collected by chorionic villus sampling, amniocentesis, or fetal blood sampling. The genetic test consists in the detection of a 1q21.1 microdeletion, which affects both alleles of the RBM8A gene^(3)^. Although there is no evidence that nuchal translucency plays a role in screening for TAR syndrome, there have been reports of increased nuchal translucency and cystic hygroma in fetuses with TAR syndrome^(5)^. The differential diagnoses of TAR syndrome include ATRUS syndrome, Holt-Oram syndrome, Roberts syndrome, Fanconi anemia, thalidomide embryopathy, and VACTERL association^(3)^. A diagnosis of TAR syndrome calls for intrauterine platelet transfusion and for planning a delivery method that will prevent peripartum bleeding^(6)^.

The treatment consists of support according to the degree of thrombocytopenia, orthopedic interventions when necessary, and the avoidance of cow's milk in the diet. Bone marrow transplantation is not necessarily indicated, given that the thrombocytopenia tends to resolve spontaneously by the time the child reaches school age. After the critical period of thrombocytopenia has passed, the evolution is favorable, although there have been reports of subsequent acute lymphoblastic and myeloid leukemia^(7)^.

In summary, TAR syndrome, albeit rare, has a very specific presentation and can be diagnosed in the prenatal period by ultrasound. The initial treatment and measures for the prevention of complications from bleeding can be started *in utero*. Certain invasive procedures also permit the genetic diagnosis of TAR syndrome to be made during pregnancy, thus making it possible to provide appropriate genetic counseling.
